# Impact of Conventional vs. Vertical Tooth Extraction on Three-Dimensional Soft Tissue Remodelling and Aesthetic Parameters of Adjacent Teeth: One-Year Results of a Randomized Clinical Trial

**DOI:** 10.3390/dj14010046

**Published:** 2026-01-12

**Authors:** Jonas Kopp, Ragai Edward Matta, Mayte Buchbender, Werner Adler, Marco Kesting, Manfred Wichmann, Anna Seidel

**Affiliations:** 1 Department of Prosthodontics, University Hospital Erlangen, Friedrich-Alexander Universität Erlangen-Nürnberg (FAU), 91054 Erlangen, Germany; jonas.kopp@uk-erlangen.de (J.K.); ragai.matta@uk-erlangen.de (R.E.M.);; 2 Department of Oral and Cranio-Maxillofacial Surgery, University Hospital Erlangen, Friedrich-Alexander Universität Erlangen-Nürnberg (FAU), 91054 Erlangen, Germany; 3 Department of Medical Informatics, Biometry and Epidemiology, Friedrich-Alexander Universität Erlangen-Nürnberg (FAU), 91054 Erlangen, Germany; werner.adler@fau.de

**Keywords:** tooth extraction, periodontal atrophy, dental papilla, gingival recession, aesthetics, three-dimensional evaluation

## Abstract

**Objectives**: Post-extraction remodelling of hard and soft tissues results in volume reduction, leading to aesthetic challenges in planning prosthetic restorations, particularly in the anterior maxilla. This study assessed whether atraumatic vertical extraction, versus conventional extraction, could reduce postoperative volume loss and aesthetic compromises at the extraction site and adjacent teeth. **Methods**: Following randomized tooth extraction with unassisted healing in the test (Benex^®^ extraction, *n* = 10) and control group (conventional extraction, *n* = 10), postoperative scans were conducted at 30 days (t_1_), 60 days (t_2_), 90 days (t_3_) and 12 months (t_4_). Each scan was aligned with the baseline scan (t_0_), and surface comparison was performed with five regions of interest (ROIs: central, mesial, distal, papilla mesial and papilla distal). Aesthetic parameters, including recession and Pink Esthetic Score (PES) of adjacent teeth, were clinically evaluated at each follow-up appointment. Statistical analysis used a mixed linear model accounting for confounding factors such as smoking, buccal bone integrity, gingival phenotype, and provisional use. **Results**: Both groups showed significant volume reduction from baseline to t_3_ and t_4_. The largest volume loss occurred in the central ROI in both test (t_4_: −65.34 ± 36.89 mm^3^) and control group (t_4_: −70.85 ± 30.96 mm^3^), with no significant difference between groups. A decline in PES and recession at the adjacent teeth was noted in both groups at 12 months. **Conclusions**: Both groups showed significant volume reduction with aesthetic impairment at the adjacent teeth’s soft tissue.

## 1. Introduction

Despite the advancements in contemporary dental materials and restorative and prosthodontic techniques designed to preserve the functionality of natural teeth for extended durations, inherent limitations persist. Once these limitations are reached, tooth extraction becomes the sole viable treatment option, rendering it one of the most frequently performed dental procedures. However, this surgical procedure has consequences—especially in the aesthetic zone. Biological remodelling and resorption processes at the site of the missing tooth lead to significant local anatomical changes in the alveolar ridge [1,2,3]. Since the most severe bone resorption occurs at the buccal plate, resulting in reduced soft tissue support in this area, there is pronounced volume loss in this region and soft tissue recession on the adjacent teeth. In quantitative terms of millimetres, a systematic review showed that, as a result of an extraction with physiological healing of the socket, there was a reduction in the alveolar ridge width of 2.6 mm to 4.6 mm and a loss of height between 0.4 mm and 3.9 mm [4]. The atrophy of the bony alveolar ridge and the recession of the buccal soft tissue on both the neighbouring teeth and the pontic area can significantly affect the prosthetic restoration following tooth extraction. This is particularly important in the aesthetic anterior region, both with conventional prosthetic restorations and with implant-supported restorations [5,6]. For this reason, the clinician should plan the procedure and restoration prior to the extraction, taking into consideration the atrophy of the alveolar ridge [7,8]. It has been shown that the less traumatic the extraction procedure, the less hard and soft tissue loss occurs in the region of the extracted tooth, thus facilitating subsequent functional and aesthetic prosthetic rehabilitation [9]. One method to achieve a less invasive extraction without significant expansion of the alveolus is the atraumatic vertical extraction system. In this approach, the necessary force for extraction is applied to the tooth by inserting a screw into the root canal along the tooth axis, allowing for extraction from the alveolar process through axial traction along the root (e.g., with the Benex^®^ extraction system) [10,11,12]. This technique minimizes invasiveness by preserving bone and soft tissue structures [10,11]. However, in addition to the extraction method, there are other factors that influence wound healing and three-dimensional tissue changes. Studies have shown that increased cigarette consumption, a thin buccal bone wall and a thin phenotype can have a negative effect on the postoperative healing process [6,13,14]. Knowledge about the behaviour of hard and soft tissue after an extraction, especially in the aesthetic zone at different stages of healing, is crucial for effective prosthetic planning and rehabilitation of the edentulous space. Consequently, this study examines the digital and three-dimensional evaluation of postoperative soft tissue changes over the course of one year, comparing vertical Benex^®^ extraction (Benex^®^, Bone Management^®^ System Benex^®^-Control, Hager & Meisinger GmbH, Neuss, Germany) with conventional extraction using lever and forceps, with particular attention to the aesthetically demanding vestibular anterior tooth region in the maxilla.

## 2. Materials and Methods

### 2.1. Study Design and Study Cohort

This prospective randomized controlled clinical trial was conducted in accordance with the Declaration of Helsinki on medical protocol and ethics and carried out after approval of the local medical ethics committee of the medical faculty of Friedrich-Alexander University Erlangen-Nuremberg, Germany (Approval no. 42_20 B, on 25 May 2020) [15]. The study was registered prior to data analysis (German clinical trial registry, DRKS00024089) and CONSORT guidelines [16] were followed for reporting (see supplementary material, File S1: CONSORT checklist). All patients gave written informed consent prior to the inclusion of their data in this trial.

This article serves as a complement and continuation of the research conducted by the same working group, with detailed specifications regarding the patient cohort available within the article of Buchbender et al. [17].

The inclusion criteria for both groups were as follows:

- Patients ≥18 years of age and in good general health;
- Patients needing tooth extraction in the aesthetic zone (13–23);
- Patients with healthy periodontium and gingiva.

The exclusion criteria for both groups were as follows:

- Inflammation of the periodontium or gingiva (periodontitis or gingivitis) at the extraction area or adjacent teeth;
- Previous mucogingival surgery (i.e., root tip resection) at the extraction area or adjacent teeth;
- Pregnant or breastfeeding patients;
- Current or previous radiotherapy of the head and neck area and/or chemotherapy;
- Systemic medication that could affect the outcome of the therapy, such as medications that can induce gingival hyperplasia (anticonvulsants, immunosuppressants, and calcium antagonists), as well as antiresorptive agents (bisphosphonates and monoclonal antibodies).

**Figure 1:**
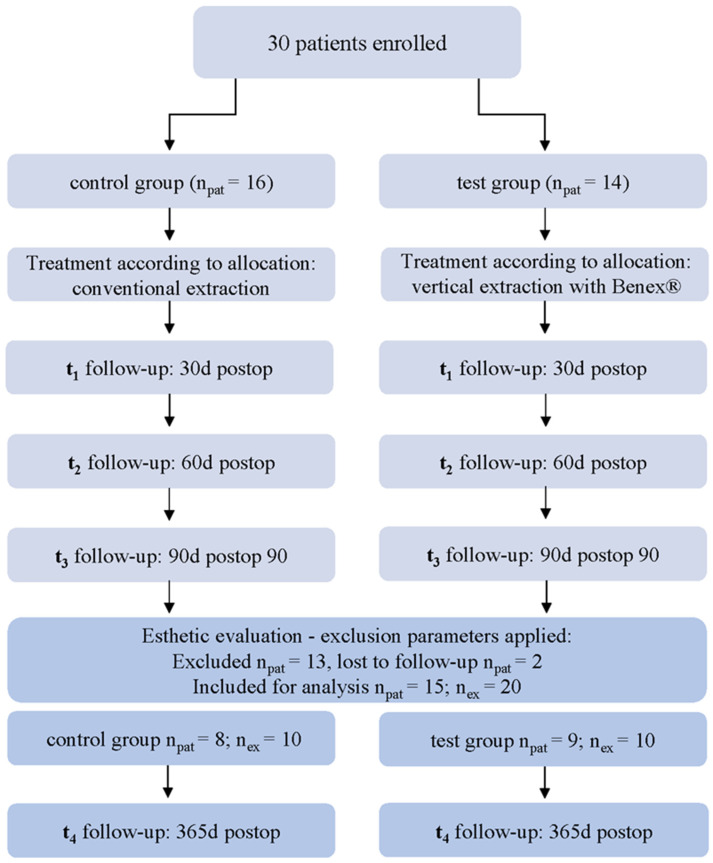
Study design flowchart and allocation overview.

In the primary study by Buchbender et al., 30 subjects were enrolled and randomly allocated in a 1:1 ratio to either the intervention (n_pat_ = 14; vertical extraction according to the Benex^®^system) or control group (n_pat_ = 16; conventional extraction) using a computer-generated randomization sequence (intention-to-treat) [17]. The allocation process was concealed using sequentially numbered, opaque, sealed envelopes that were opened after enrolment, prior to surgery. In accordance with the aim of this 12-months follow-up analysis to examine soft tissue remodelling at the extraction site and the aesthetic parameters of adjacent teeth, it was restricted to participants in the original randomized controlled trial who fulfilled an additional, pre-specified anatomical criterion, namely the presence of two adjacent teeth next to the region of interest. Of the 30 patients randomized in the parent trial, 13 did not meet this criterion and were therefore not evaluable for the present outcome (Figure 1), although they remained part of the main trial cohort.

Additionally, two patients were lost to follow-up, with one being unreachable and the other deceased. The final data collection was conducted on a cohort of 15 patients, encompassing a total of 20 extraction sites (n_ex_ = 20; patients with split-mouth treatment *n* = 4; Figure 1). Of these, ten teeth were extracted using conventional methods (control group, n_ex_ = 10), while the remaining ten were extracted using the Benex^®^ extraction system (test group, n_ex_ = 10). The authors conducted an examination of this patient cohort over extended follow-up periods of up to 12 months, during which additional parameters that had not been previously described within the same cohort were assessed. Specifically, soft tissue volume at the extraction site, papilla volume, Pink Esthetic Score (PES), Recession Type (RT) classification, and measured absolute values of recession of teeth adjacent to the extraction site over a 1-year follow-up period after surgical intervention were assessed. As this publication is a continuation of the research by the same team, details regarding patient selection, surgical procedures, and methodology were comprehensively described in the previous publication [17].

### 2.2. Primary Outcome Variable

#### Volumetric Outcome Evaluation

- ΔVolume (average changes in volume): measured within a defined ROI at the STL models from intraoral scans at baseline (t_0_) superimposed with model scans 30 days (t_1_), 60 days (t_2_), 3 months (t_3_) and 12 months (t_4_) follow-up for the test and the control group; in cubic millimeters [mm^3^].

### 2.3. Secondary Outcome Variables

#### 2.3.1. ΔVolume in Correlation with Possible Confounding Variables

The secondary outcome was defined as the intergroup difference, assessed using a mixed linear model with the relative volume change in mm^3^ as the dependent variable. The analysis considered the potential influence of the following variables:

- Gingival phenotype (thin; medium; thick) [18];
- Morphology of the buccal bone/labial cortical plate (intact; fenestrated; fractured);
- Smoking behavior (none; <10 cig/d; ≥10 cig/d);
- Application of a provisional prosthesis (yes; no).

#### 2.3.2. Aesthetic Outcome Evaluation

To determine whether tooth extraction and the subsequent remodelling of supporting tissue, such as alveolar bone and soft tissue, affect not only the extraction site but also the adjacent teeth in the aesthetic region, the following parameters were evaluated to assess aesthetic outcomes of the teeth next to the extraction site:

- Recession (absolute value): Measurement of gingival recession that occurred at the buccal aspect of the adjacent teeth mesial and distal to the ROI (extraction site), defined as distance from the level of the marginal gingiva at baseline (t_0_) and the 12-month follow-up (t_4_) in millimeters [mm].
- RT (recession type): Classification of the recession type according to Cairo’s classification RT1–RT3, recorded at the buccal aspect of the adjacent teeth mesial and distal to the ROI (extraction site) at baseline (t_0_) and at 12 months (t_4_) [19].
- PES (Pink Esthetic Score): Index according to Fürhauser et al. was applied for the objective aesthetic outcome assessment regarding soft tissue at baseline (t_0_) compared to the value obtained at the final follow-up, one year post-extraction (t_4_); addressing papillae, curvature and level of facial mucosa, soft tissue color and texture [20,21].

All clinical measurements were taken by one calibrated and trained examiner.

### 2.4. Treatment Procedure

Each extraction was performed under local anaesthesia (Ultracain^®^ DS; adrenaline 1:200,000; Sanofi-Aventis GmbH, Frankfurt, Germany). All extractions were performed by an experienced oral surgeon at the Department of Oral and Maxillofacial Surgery, who has previously been trained in the use of the Benex^®^ extraction system. In the control group, the tooth was extracted as gently as possible using a lever and forceps, and in the test group the vertical extraction using the Benex^®^ system. The extraction socket was treated by adapting the wound edges using a cross suture with Vicryl^®^ 5-0 Rapide (Ethicon GmbH & Co KG, Norderstedt, Germany) in order to stabilize the coagulum after extraction. In both groups, no additional materials were applied to the alveoli, and healing proceeded without intervention. Each patient was then informed about postoperative care [17].

### 2.5. Digital 3D Data Evaluation

**Figure 2:**
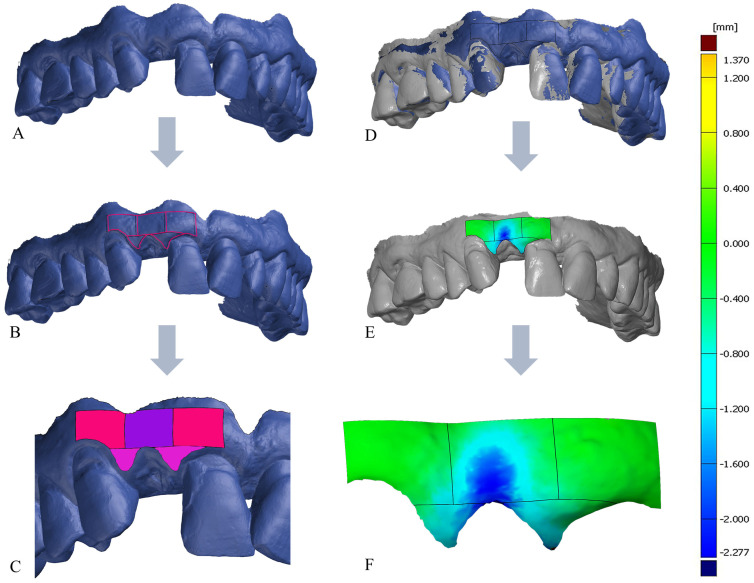
(**A**): baseline scan (t_0_); (**B**): baseline scan (t_0_) with defined ROIs; (**C**): Illustration of the ROIs; Central ROI: purple, mesial and distal ROI: magenta, papilla mesial and distal ROI: pink; (**D**): baseline scan (t_0_) (blue) and postoperative scan (t_2_) (grey) aligned; (**E**): postoperative scan (t_2_) with surface comparison; (**F**): surface changes in the defined ROIs in color-coded distance map; volume decreases are shown in blue and no changes in green.

The intraoral scans taken at the various examination times served as the basis for the digital evaluation. The digital analysis was conducted through a surface comparison between the preoperative baseline scan (t_0_), serving as the reference, and the respective postoperative follow-up scans (t_1_, t_2_, t_3_ and t_4_). The baseline scan (t_0_) was imported into the 3D analysis software GOM Inspect V 2018 (GOM Inspect, GOM GmbH, Braunschweig, Germany) and subsequently trimmed. To ensure consistent evaluation, the areas for comparison were delineated on the baseline scans (t_0_) according to a predetermined scheme. These areas comprised five defined regions: the vestibular surfaces of the papillae both mesial and distal to the tooth scheduled for extraction, as well as three surfaces in the vestibular region of the tooth to be extracted. The definition of these surfaces was accomplished by constructing planes in the approximal area of the tooth to be extracted and then shifting these planes mesially and distally in parallel by the distance of the interproximal gap of the tooth to be extracted. The horizontal boundaries were established by placing a plane along the deepest point in the vestibular sulcus of the tooth to be extracted and then shifting this plane five millimetres apically, in parallel. By employing cuts along the constructed planes, the five surfaces (mesial, central, distal, mesial papilla, and distal papilla) were isolated as distinct regions for analysis (Figure 2). The ROIs are defined by the following anatomic landmarks:

- Central ROI: at the vestibular aspect of the tooth to be extracted starting apical to the sulcus for 5 mm with borders at the approximal contacts of the adjacent teeth.
- Mesial/Distal ROI: Same width as the Central ROI, located directly mesial and distal to it, apical the sulcus of the mesial and distal adjacent teeth.
- Papilla mesial and Papilla distal ROI: area above the mesial, distal, and central ROIs in shape of the papillae of the tooth to be extracted and the respective mesial and distal adjacent teeth.

Subsequently, the baseline scan with the five defined comparison surfaces was converted into a CAD model. For volumetric comparison, each postoperative scan (t_1_, t_2_, t_3_, and t_4_) was imported separately into the analysis software. The CAD model of the baseline scan and the respective postoperative scan were meticulously aligned using a local best-fit based on the adjacent mesial and distal neighbouring teeth [22]. Utilizing the analysis software GOM Inspect, a surface comparison was generated for each defined area. This process yielded the volume increases or decreases in cubic millimetres [mm^3^] from the baseline scan to the respective follow-up scan for the five surfaces [23] (Figure 2). The reliability of the evaluation was ensured by the fact that the digital evaluation was carried out by the same trained and experienced evaluator using a well described and defined protocol and that the calculations of the volume change were purely software and algorithm-based.

### 2.6. Statistical Analysis

Statistical analysis was performed using the statistical software R V4.3.1 (R Core Team 2023, R: A language and Environment for Statistical Computing, Vienna, Austria) [24], by a blinded statistician. The analysis of volume change was conducted by considering the potential influence of confounding factors through the application of a mixed linear model (simple random intercept models, i.e., repeated measures are handled by assuming the same intercept for each patient in the study). The relative volume change, serving as the dependent variable, was modelled using several independent variables: group (control group as the reference), area (central ROI as the reference), and time (t_1_ as the reference), with only one additional variable considered in each instance. In four additional models, one of phenotype, buccal bone, provisional, and smoking behaviour served as additional independent variable. A statistical comparison of the volume across individual investigated areas (central ROI, mesial ROI, distal ROI, papilla mesial ROI, and papilla distal ROI) was performed using paired *t*-tests for comparisons of time points within the intervention groups, and independent *t*-tests for comparisons between groups at a specific follow-up time point. Missing data were not imputed. The significance level was set at *p* < 0.05 and *p*-values were adjusted for multiple testing using the Benjamini–Hochberg method.

## 3. Results

### 3.1. Patient Demographics

The final data collection was conducted on a cohort of 15 patients, encompassing a total of 20 extraction sites (Table 1). Of these, ten teeth were extracted using conventional methods (control group, n_ex_ = 10; n_pat_ = 8), while the remaining ten were extracted using the Benex^®^ extraction system (test group, n_ex_ = 10; n_pat_ = 9). Healing of all extraction sockets proceeded without complications in both the test and control groups and no adverse events occurred during the study period.

### 3.2. 3D Volumetric and Linear Evaluation at ROI

**Figure 3:**
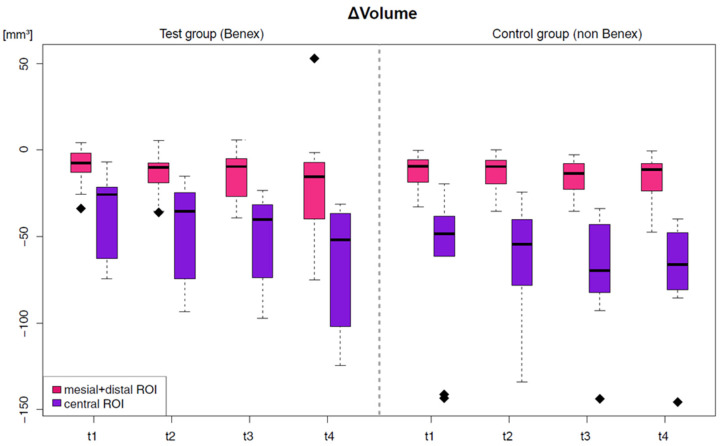
Comparative analysis of the volumetric changes [mm^3^] depicted as boxplots—central ROI (purple) and the combined mesial ROI and distal ROIs (pink) at the follow-up intervals t_1_, t_2_, t_3_, and t_4_ for the test and control group. Outliers are represented as rhombuses.

The change in volume was documented for all areas defined as described above at the respective examination times, relative to the preoperative baseline volume. Table 2 presents the results of the digital evaluation in cubic millimeters. The most relevant volume reduction throughout the study period was observed in the central ROI compared to all other ROIs examined (Figure 3). The smallest mean volume reduction in the central region occurred in the test group at the first postoperative follow-up (t_1_), measuring −37.89 ± 23.50 mm^3^. The most pronounced volume loss was recorded in the control group at the final follow-up (t_4_), one year post-extraction, with −70.85 ± 30.96 mm^3^. The evaluation of volume change in the mesial and distal ROIs indicated a volume decrease in both groups at each examination time point. In contrast to the other two vestibular ROIs, the volume in the distal ROI did not decrease continuously. Regarding the volume-to-surface ratio [mm^3^/mm^2^] in the central ROI, both groups showed a decrease in volume over the entire study period, which was more pronounced in the control group at every point of measurement (Table 2).

### 3.3. ΔVolume and Possible Confounding Variables: Mixed Linear Model

To address potential confounding variables and enable comparisons among individual groups, the analysis employed a mixed linear model. In this model, the relative volume change in mm^3^ was designated as the dependent variable, with group, area, and time serving as independent variables. The control group served as the reference for the group variable, the central ROI was the reference for the area variable and time point t_1_ was the reference for the time variable. Within this mixed linear model, only the variables at time point t_3_, with a *p*-value of 0.01, and time point t_4_, with a *p*-value of 0.013, demonstrated statistical significance compared to t_1_ (Table 3). These remained the only significant differences even after adjusting for phenotype, buccal lamella, temporary restoration, and smoking behaviour. Between time points t_1_ and t_3_, a significant relative volume change of 47.98% was observed. Similarly, over the period from time point t_1_ to t_4_, a significant relative volume change of 44.52% was noted.

### 3.4. Aesthetic Parameter Analysis

**Figure 4:**
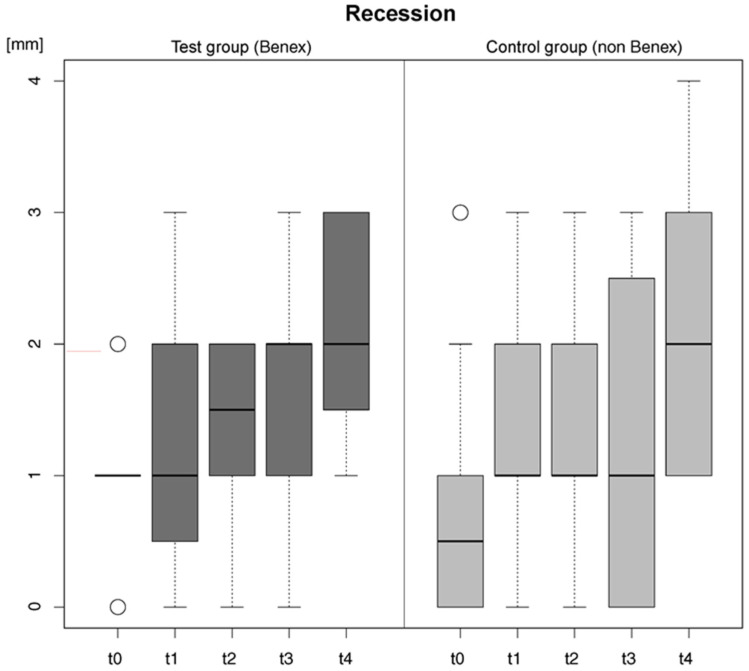
Box plots illustrate the measurements of recession of the teeth adjacent to the region of interest (ROI) both mesially and distally, expressed in millimeters, at the respective examination intervals t_0_, t_1_, t_2_, t_3_, and t_4_ for the test group (dark grey) and the control group (light grey). Outliers are represented as white circles.

The assessment of gingival recession in the mesial neighbouring teeth adjacent to the extracted tooth throughout the study period demonstrated a consistent increase in recession measurements from the baseline (t_0_) to the final time point (t_4_) for both intervention groups (Table 4). The most relevant recession was observed at the final time point (t_4_), one year postoperatively, with the control group exhibiting 2 ± 0.86 mm mesially and 2.25 ± 0.97 mm distally, and the test group showing 2.11 ± 0.74 mm mesially and 2.00 ± 0.82 mm distally. The mean initial recession (values for mesial and distal in total) at time t_0_ was 0.83 ± 1.02 mm for the control group and 1.08 ± 0.49 mm for the test group, after one year at t_4_, the mean recession was 2.2 ± 0.94 mm for the control group and 2.08 ± 0.79 mm for the test group (Figure 4). The mean change in recession from t_0_ to t_4_ observed on the adjacent teeth throughout the study was 1.37 ± 1.39 mm for the control and 1.00 ± 0.93 mm for the test group. In both groups, the most substantial increase in recession occurred between the penultimate examination time point (t_3_) and the endpoint (t_4_).

To assess alterations in the aesthetic parameters of the teeth adjacent to the region of interest (ROI), the PES was employed throughout the study. The baseline value at the preoperative time point t_0_ was compared with the value at the final time point one year post-extraction t_4_. The findings indicated a decrease in the PES over the study period, irrespective of the group or variable. On average, the PES declined from 11.0 ± 0.9 to 7.5 ± 1.5 (Table 4). A comparison between the two groups revealed a more pronounced decrease in the test group (4.5) compared to the control group, where the PES decreased by 2.6 over the study duration. When considering phenotype as a variable, there was only a slight difference in the decrease in PES at time t_4_. The evaluation of the RT classification on the adjacent teeth of the extraction sockets showed, as already seen in the evaluation of recession in mm, an increase over the course of the study (Table 4). No significant difference between the groups was detected.

## 4. Discussion

The present study investigated the volumetric changes associated with aesthetic alterations following tooth extraction in the anterior aesthetic zone. In this context, the conventional extraction method (control group) was compared with an alternative method—atraumatic vertical extraction using the Benex^®^ system (test group). In both cohorts, a continuous reduction in tissue volume was observed in the buccal area of the aesthetic zone throughout the postoperative observation time of 12 months. No significant difference in volume loss was detected between the groups, although there was a tendency for reduced volume loss in the test group. This slight difference may be attributed to the fact that conventional extractions were also performed in an atraumatic manner by an experienced oral surgeon, without the need for flap formation or osteotomy. The findings of Buchbender et al. similarly indicated no significant difference between conventional and Benex^®^ extractions concerning volumetric changes at 6 months [17]. Significant changes in volume loss were noted at 90 days (t_3_) and one year postoperatively (t_4_). On average, the central region of interest in the control group exhibited a volume change of −70.85 ± 30.96 mm^3^, while the test group showed a change of −65.34 ± 36.89 mm^3^ one year post-extraction (t_4_)—which corresponds to a linear tissue loss of −1.94 ± 0.61 and −1.88 ± 0.77 mm, respectively. According to this study, postoperative volume changes can occur over an extended period following extraction. This finding suggests that, in clinical practice, a sufficiently prolonged interim or temporary phase is advisable before the insertion of the final dental prosthesis, particularly in the aesthetically significant anterior region. Premature insertion of the final restoration, amidst ongoing tissue remodelling, may lead to recessions, resulting in aesthetic impairments. In a worst-case scenario, if, i.e., the basal element of a tooth-supported fixed dental prosthesis no longer fits satisfactorily or if cervical areas become exposed, the prosthesis may need to be remade. This volume loss likely compromises the natural aesthetic appearance of the alveolar ridge in the vestibular aspect, as similarly noted in the study conducted by Chenchev et al. [25]. This was corroborated by the decrease in the PES over the study period in both groups, influenced by factors such as the buccal contour of the alveolar ridge, the height of the gingival margin, and the height of the papillae [21,26]. The three-dimensionally observed volume reduction in the ROI of the adjacent papillae further confirmed the diminished aesthetics. In this study, the use of a provisional restoration postoperatively did not significantly enhance the preservation of the papillae. Bakshi et al. and Yang et al. reported in their study, that the use of a fixed ovate pontic provisional placed immediately after tooth extraction leads to a reduced change in width and height of the alveolar ridge [27,28]. Yang et al. even found, that the preservation of the gingival papilla was more pronounced in cases with immediate pontic provisional restoration [28]. Nonetheless, Bakshi et al. and Yang et al. reported a significantly reduced volume loss with the use of a provisional restoration. This discrepancy may be attributed to the inclusion of both fixed and removable provisionals in the current study, as opposed to the exclusive use of fixed provisionals in the comparative studies. It is plausible that the tissue-supporting effect is substantially compromised when removable provisionals are employed. The majority of current studies on provisional dentures post-extraction relate to implant-supported dentures and are therefore difficult to compare with this study. Unfortunately, there is very little current data available for comparable studies. However, several studies indicate that the use of implants and implant-supported dentures following tooth extraction can support the surrounding tissue and prevent resorption, thereby positively influencing the Pink Esthetic Score [29,30,31,32].

Overall, potential confounding factors did not appear to significantly influence volumetric changes following tooth extraction in this study. This phenomenon may be attributed to the limited patient cohort, and the fact that the volume loss following extraction was so pronounced that cofactors exerted only a minimal influence. It can be assumed that these variables may have a more relevant influence in larger cohorts, as other studies have shown [13,14]. Therefore, there is a need for multicenter, large-scale studies that systematically take such risk factors into account. In the study by Makki et al., postoperative pain perception and the healing process of the extraction socket following conventional extraction were compared with extraction using the Benex^®^ system [33]. Although volumetric changes post-extraction were not examined, four weeks post-extraction, the Benex^®^ group demonstrated excellent wound healing in 42.1% of cases compared to 5.3% in the control group. Studies suggest that extraction with the Benex^®^ system was less traumatic, causing less damage to surrounding structures, potentially resulting in reduced volume loss [25,33]. The discrepancy in the findings suggests that the advantages of the Benex^®^ system are particularly evident in clinical situations where conventional techniques are associated with a higher risk of soft tissue and bone loss [25]. In our collective, however, all extractions were performed atraumatically by experienced surgeons, which may have levelled out the difference.

The digital virtual three-dimensional analysis utilizing 3D models constitutes a highly precise methodology and is recognized as an established approach for conducting analyses over extended periods [23]. This technology facilitates the assessment of volumetric changes across entire surfaces, as opposed to isolated point measurements typically obtained using instruments such as probes or endodontic files under anaesthesia [34]. It is important to note that tissue remodelling post-extraction occurs over a broader area rather than at a singular point. Consequently, purely linear measurements would have been inadequate for the study’s objectives. Given that the regions of interest (ROI) in this study were delineated according to a standardized scheme but exhibited slight variations due to patient-specific anatomical differences, the volume-to-area ratio was calculated to enhance comparability. Similar values were observed for the area located vestibular-apical to the extraction socket, identified as ROI 3 in Buchbender et al., which corresponds to the central ROI in this study. In Buchbender et al. [17], an average reduction of −1.59 mm in the control group and −1.39 mm in the test group was reported in this region, whereas the present study recorded an average reduction of −1.84 mm in the control group and −1.43 mm in the test group after 90 days.

This study exclusively focused on the extraction of single-rooted maxillary anterior teeth, as these are optimally suited for the vertical extraction. In studies incorporating multi-rooted teeth, the Benex^®^ extraction system demonstrated certain limitations in its application. In the clinical study by Muska et al. for some cases, it was necessary to create a flap and remove alveolar bone to facilitate the procedure. This approach inevitably results in a prolonged healing process and more significant volume loss [11]. The Benex^®^ extraction system demonstrated a notably high success rate in clinical applications within this study, a finding corroborated by other research involving larger patient cohorts. Specifically, in the study conducted by Hong et al., which encompassed 323 tooth extractions, 85.4% of the teeth were successfully extracted utilizing the Benex^®^ system [10].

Even though this study did not find any significant difference between conventional extraction and Benex^®^ extraction, there are studies that show the Benex^®^ extraction system to be a useful implementation in everyday clinical practice. Chenchev et al. and Makki et al. both showed reduced postoperative pain in patients after vertical extractor use compared to conventional extraction. Furthermore, postoperative wound healing was found to be improved in both studies by the use of the Benex^®^ system and was associated with fewer complications [25,33]. The Benex system has demonstrated its suitability for planned immediate implantation, primarily due to its potential for tissue preservation [35]. In terms of operating time, when experienced with the Benex^®^ system, no additional time requirement was found for a Benex^®^ extraction compared to a conventional extraction [17].

This study has some limitations that need to be addressed. Primarily, the conclusions were drawn from a small sample size and therefore, a cautious interpretation of the results is necessary. Furthermore, some measurements, such as gingival thickness or recession, were taken manually with a probe, thus introducing possible bias, even though the examiner was well trained and calibrated. Another significant drawback of this study is that the digital evaluation was based on a surface comparison of the obtained intraoral scans and therefore does not allow for differentiation between the loss of bone or soft tissue volume. To fully consider this fact, as conducted in other studies, a 3D-radiographical image would have been necessary for differentiation [36,37]. This was omitted from this study, as additional ionizing radiation exposure of the patients solely for the purposes of this study would not have been ethically justifiable. The evaluation in this study reflects the clinical results, that is, the visual postoperative situation of the patients, with which the dentist must contend for rehabilitation treatment. Chappuis et al. demonstrated that following an extraction, both the alveolar bone and adjacent soft tissue undergo remodelling; however, the reduction in bone structures accounts for the greater share of volume loss [37]. For the volume changes found in this study, it can therefore be assumed that they result from a combination of remodelling processes in both bone and soft tissue.

There are already promising studies that have investigated the preservation of volume after tooth extraction using various methods of alveolar ridge preservation. The results of different systematic reviews indicated that the postoperative filling of the socket with bone graft is an effective therapy to reduce physiological bone loss after extraction [38,39,40]. Therefore, it could be beneficial in the future to conduct further studies on volume preservation of the tissue after tooth extraction, especially as patients’ aesthetic demands increase. The digital evaluation method employed in this study is of significant value due to its high reproducibility and its capacity to accurately detect even the smallest changes in volume.

## 5. Conclusions

Following both conventional and vertical tooth extraction interventions in the upper aesthetic zone, a reduction in soft tissue was noted over the 12 months observation time in the vestibular area of the extracted tooth, affecting both central volume and papillae. A change in the recession of the teeth adjacent to the extraction socket was observed up to one year postoperatively. Although the vertical extraction system was associated with a marginally lower volume loss and a less reduced Pink Esthetic Score during the study period, this difference did not reach statistical significance. Understanding the volumetric and aesthetic changes following tooth extraction is essential, as these alterations can affect the intended aesthetic prosthodontic rehabilitation.

## Figures and Tables

**Table 1:** Patient demographics detailing the allocation into the test group or the control group, along with a list of the respective observation parameters.

Parameters		Number (n_ex_)	Group	Number (n_ex_)
Sex	Female	7	Control	3
			Test	6
	Male	8	Control	7
			Test	4
Teeth	Total	20	Control	10
			Test	10
Tooth	Primary incisor	4	Control	1
			Test	3
	Lateral incisor	11	Control	5
			Test	6
	Canine	5	Control	4
			Test	1
Reason for extraction	Non-restorable carious lesions	18	Control	8
			Test	10
	High mobility	1	Control	1
			Test	0
	Fracture along longitudinal axis	1	Control	1
			Test	0
Age (Mean ± SD)	Total	65.3 ± 11.2	Control	63.9 ± 12.0
			Test	65.2 ± 10.1
Smoking	Non-smoker	10	Control	7
			Test	6
	≤10 Cig./day	2	Control	1
			Test	2
	>10 Cig./day	3	Control	2
			Test	2
Provisional	yes	10	Control	7
			Test	3
	no	10	Control	3
			Test	7
Gingival Phenotype	thin	4	Control	2
			Test	2
	medium	5	Control	2
			Test	3
	thick	11	Control	6
			Test	5
Buccal bone	intact	12	Control	5
			Test	7
	fenestrated	2	Control	1
			Test	1
	fractured	6	Control	4
			Test	2

**Table 2:** Results of volume changes within the central, mesial, and distal ROIs, as well as for the ROIs of the mesial and distal papillae, in both the control and test groups at follow-up examination time points t_1_–t_4_. Furthermore, for ROI central, results of the ratio of volume to surface area in mm^3^/mm^2^ are given. The rows of the test group are shaded in grey to enhance readability. The table includes calculated arithmetic mean values (Mean) with corresponding standard deviations (SD), along with the largest (Max) and smallest (Min) observed values.

**Follow-Up**	**Group**	**ΔVolume**											
		**Central ROI**				**Mesial ROI**				**Distal ROI**			
		**Mean [mm^3^]**	**SD [mm^3^]**	**Min [mm^3^]**	**Max [mm^3^]**	**Mean [mm^3^]**	**SD [mm^3^]**	**Min [mm^3^]**	**Max [mm^3^]**	**Mean [mm^3^]**	**SD [mm^3^]**	**Min [mm^3^]**	**Max [mm^3^]**
t_1_	Control	−62.57	±43.87	−143.34	−19.42	−13.6	±10.63	−32.82	−0.21	−10.55	±9.79	−31.03	−0.63
	Test	−37.89	±23.5	−74.38	−6.90	−7.22	±9.86	−25.49	4.16	−11.17	±11.23	−33.8	1.99
t_2_	Control	−62.78	±32.93	−133.93	−24.29	−14.24	±10.85	−35.48	0.08	−9.94	±7.98	−22.89	−0.2
	Test	−46.65	±27.15	−93.52	−15.25	−9.37	±10.0	−28.56	5.38	−21.18	±11.65	−36.03	−7.44
t_3_	Control	−70.02	±33.11	−143.75	−33.96	−16.97	±10.03	−35.55	−3.95	−13.56	±10.1	−31.57	−2.78
	Test	−51.46	±25.04	−97.28	−23.22	−11.23	±12.46	−29.47	5.85	−20.26	±14.44	−39.33	−4.86
t_4_	Control	−70.85	±30.96	−145.63	−39.78	−21.98	±16.91	−47.38	−0.90	−11.04	±7.89	−27.08	−0.47
	Test	−65.34	±36.89	−124.34	−31.13	−25.99	±23.23	−74.88	−1.36	−12.53	±33.78	−48.69	52.91
		**ΔVolume**								**ΔVolume/Surface Area [mm^3^/mm^2^]**			
		**Papilla Mesial ROI**				**Papilla Distal ROI**				**ROI Central**			
		**Mean [mm^3^]**	**SD [mm^3^]**	**Min [mm^3^]**	**Max [mm^3^]**	**Mean [mm^3^]**	**SD [mm^3^]**	**Min [mm^3^]**	**Max [mm^3^]**	**Mean [mm]**	**SD [mm]**	**Min [mm]**	**Max [mm]**
t_1_	Control	−12.10	±7.68	−24.71	−3.09	−11.41	±8.50	−28.75	−2.03	−1.65	±0.80	−3.26	−0.67
	Test	−7.82	±3.97	−14.35	−1.59	−10.66	±5.31	−20.52	−5.93	−1.03	±0.47	−1.87	−0.30
t_2_	Control	−12.91	±8.14	−26.30	−4.29	−9.70	±5.84	−18.38	−1.95	−1.75	±0.82	−3.43	−0.82
	Test	−10.03	±3.77	−16.03	−2.60	−15.64	±7.83	−30.05	−7.20	−1.30	±0.52	−2.23	−0.48
t_3_	Control	−13.79	±7.42	−26.30	−5.34	−10.78	±6.32	−20.83	−2.59	−1.84	±0.72	−3.45	−1.00
	Test	−10.09	±5.36	−20.37	−2.68	−15.03	±7.66	−28.34	−6.81	−1.43	±0.38	−1.99	−1.00
t_4_	Control	−15.63	±11.00	−32.92	−5.01	−10.19	±6.31	−20.89	−1.99	−1.94	±0.61	−3.36	−1.24
	Test	−13.72	±8.36	−30.20	−3.26	−17.24	±10.90	−35.18	−2.81	−1.88	±0.77	−3.72	−1.01

**Table 3:** Mixed linear model with relative volume change as the dependent variable, independent variables calculated are: intervention group (reference: control group); area (ref.: central area) and time (ref.: time t_1_) for gingival phenotype (ref.: thin), buccal bone (ref.: fractured), use of provisional (ref.: no provisional), smoking behaviour (ref.: non-smoker). The values of the coefficient and 95% confidence interval (95% CI) reflect the percentage changes. The *p*-value is statistically significant for values ≤ 0.05.

Variable	Coefficient	95% CI	*p*-Value
**ΔVolume base model**			
Test group	−6.522	−49.71; 36.666	0.771
ROI distal	−12.279	−59.225; 34.666	0.609
ROI mesial	13.99	−31.204; 59.185	0.544
ROI Papilla distal	−23.473	−69.821; 22.875	0.322
ROI Papilla mesial	−7.301	−52.488; 37.885	0.752
t_2_	13.601	−27.445; 54.646	0.516
t_3_	54.591	13.545; 95.636	**0.01**
t_4_	52.159	11.113; 93.204	**0.013**
**Phenotype**			
Phenotype medium	13.548	−54.996; 82.092	0.704
Phenotype thick	11.949	−47.709; 71.608	0.7
Test group	−6.72	−52.631; 39.191	0.778
ROI distal	−11.744	−58.728; 35.239	0.625
ROI mesial	13.945	−31.26; 59.149	0.546
ROI Papilla distal	−24.036	−70.414; 22.343	0.31
ROI Papilla mesial	−7.223	−52.419; 37.973	0.754
t_2_	13.602	−27.444; 54.648	0.516
t_3_	54.592	13.546; 95.638	**0.01**
t_4_	52.16	11.114; 93.206	**0.013**
**Buccal bone**			
Buccal bone intact	−17.028	−68.639; 34.582	0.527
Buccal bone fenestrated	−25.717	−107.943; 56.51	0.549
Test group	−3.373	−49.517; 42.772	0.888
ROI distal	−12.271	−59.236; 34.693	0.609
ROI mesial	14.471	−30.738; 59.68	0.531
ROI Papilla distal	−23.912	−70.28; 22.455	0.313
ROI Papilla mesial	−7.165	−52.355; 38.026	0.756
t_2_	13.65	−27.393; 54.693	0.515
t_3_	54.64	13.597; 95.683	**0.009**
t_4_	52.208	11.165; 93.251	**0.013**
**Provisional**			
Provisional yes	4.914	−43.515; 53.342	0.845
Test group	−4.553	−52.997; 43.891	0.856
ROI distal	−12.045	−59.005; 34.916	0.616
ROI mesial	14.003	−31.195; 59.201	0.544
ROI Papilla distal	−23.74	−70.108; 22.628	0.316
ROI Papilla mesial	−7.312	−52.501; 37.877	0.751
t_2_	13.592	−27.453; 54.638	0.517
t_3_	54.583	13.537; 95.628	**0.01**
t_4_	52.151	11.105; 93.196	**0.013**
**Smoking**			
<10 Zig/d	−8.912	−75.236; 57.412	0.796
≥10 Zig/d	−13.108	−71.311; 45.095	0.665
Test group	−5.647	−51.557; 40.263	0.813
ROI distal	−12.214	−59.208; 34.781	0.611
ROI mesial	14.204	−31.002; 59.41	0.538
ROI Papilla distal	−23.562	−69.932; 22.809	0.32
ROI Papilla mesial	−7.647	−52.872; 37.578	0.741
t_2_	13.559	−27.491; 54.61	0.518
t_3_	54.549	13.499; 95.6	**0.01**
t_4_	52.117	11.067; 93.168	**0.013**

**Table 4:** Aesthetic parameter evaluation. The absolute change in recession, measured on the mesial and distal aspects of the tooth adjacent to the region of interest (ROI) at baseline and during subsequent follow-up examinations (t_0_–t_4_), is presented for both the test and control groups. The rows of the test group are shaded in grey to enhance readability. The table also provides the calculated arithmetic mean values (Mean) along with the respective standard deviation (SD), as well as the maximum (Max) and minimum (Min) values, all expressed in millimeters (mm). representation of the PES at time t_0_ (preoperative, baseline) and t_4_ (12 months post-extraction) is provided for both the test and control groups, as well as for the three distinct gingival phenotypes (thin, medium, thick). The PES is presented as an arithmetic mean accompanied by the respective standard deviation. The RT classification is given at time t_0_ (before surgery, baseline value) and t_4_ (12 months after extraction), with a classification ranging from RT 1 to RT 3.

**Follow-Up**	**Group**	**Recession**							
		**Recession Mesial**				**Recession Distal**			
		**Mean [mm]**	**SD [mm]**	**Max [mm]**	**Min [mm]**	**Mean [mm]**	**SD [mm]**	**Max [mm]**	**Min [mm]**
t_0_	Control	0.89	±0.994	3	0	0.77	±1.03	3	0
	Test	1.00	±0.471	2	0	1.25	±0.433	2	1
t_1_	Control	1.14	±0.639	2	0	1.50	±0.866	3	0
	Test	1.00	±0.816	2	0	2.00	±0.816	3	1
t_2_	Control	1.29	±0.699	2	0	1.62	±0.992	3	0
	Test	1.22	±0.786	2	0	1.67	±0.471	2	1
t_3_	Control	1.29	±1.277	3	0	1.38	±1.111	3	0
	Test	1.56	±0.956	3	0	2.00	±0.816	3	1
t_4_	Control	2.00	±0.857	3	1	2.25	±0.968	4	1
	Test	2.11	±0.737	3	1	2.00	±0.816	3	1
		**PES**					**RT**		
		**Mean [mm]**	**SD [mm]**	**Variable**	**Mean [mm]**	**SD [mm]**	**Median**	**Max**	**Min**
t_0_	Total	11.0	±0.9	Phenotype thin	11.3	±0.5	1	3	0
	Control	10.6	±0.8	Phenotype medium	11.4	±1.1	1	3	0
	Test	11.4	±0.8	Phenotype thick	10.7	±0.9	1	3	1
t_4_	Total	7.5	±1.5	Phenotype thin	7.5	±2.6	2	3	1
	Control	8.0	±1.8	Phenotype medium	7.8	±1.1	2	2	1
	Test	6.9	±1.0	Phenotype thick	7.27	±1.3	2	3	1

## Data Availability

All data generated or analyzed during this study are included in this published article. Further requests can be addressed to the corresponding author.

## Supplementary Materials

The following supporting information can be downloaded at: https://www.mdpi.com/article/10.3390/dj14010046/s1, File S1: CONSORT checklist.
